# Filtering of false positive microRNA candidates by a clustering-based approach

**DOI:** 10.1186/1471-2105-9-S12-S3

**Published:** 2008-12-12

**Authors:** Wing-Sze Leung, Marie CM Lin, David W Cheung, SM Yiu

**Affiliations:** 1 Department of Computer Science, The University of Hong Kong, Pokfulam Road, Hong Kong; 2 Department of Chemistry, Open Laboratory of Chemical Biology, The University of Hong Kong, Pokfulam Road, Hong Kong

## Abstract

**Background:**

MicroRNAs are small non-coding RNA gene products that play diversified roles from species to species. The explosive growth of microRNA researches in recent years proves the importance of microRNAs in the biological system and it is believed that microRNAs have valuable therapeutic potentials in human diseases. Continual efforts are therefore required to locate and verify the unknown microRNAs in various genomes. As many miRNAs are found to be arranged in clusters, meaning that they are in close proximity with their neighboring miRNAs, we are interested in utilizing the concept of microRNA clustering and applying it in microRNA computational prediction.

**Results:**

We first validate the microRNA clustering phenomenon in the human, mouse and rat genomes. There are 45.45%, 51.86% and 48.67% of the total miRNAs that are clustered in the three genomes, respectively. We then conduct sequence and secondary structure similarity analyses among clustered miRNAs, non-clustered miRNAs, neighboring sequences of clustered miRNAs and random sequences, and find that clustered miRNAs are structurally more similar to one another, and the *RNAdistance *score can be used to assess the structural similarity between two sequences. We therefore design a clustering-based approach which utilizes this observation to filter false positives from a list of candidates generated by a selected microRNA prediction program, and successfully raise the positive predictive value by a considerable amount ranging from 15.23% to 23.19% in the human, mouse and rat genomes, while keeping a reasonably high sensitivity.

**Conclusion:**

Our clustering-based approach is able to increase the effectiveness of currently available microRNA prediction program by raising the positive predictive value while maintaining a high sensitivity, and hence can serve as a filtering step. We believe that it is worthwhile to carry out further experiments and tests with our approach using data from other genomes and other prediction software tools. Better results may be achieved with fine-tuning of parameters.

## Background

MicroRNAs (miRNAs) are small non-coding RNA gene products of 19–25 nucleotides (nt) long, which function to repress the translation or mediate the degradation of their target mRNAs. A 22 nt mature miRNA is derived from a precursor transcript of 60–80 nt in length, which is named as pre-miRNA. Pre-miRNAs can potentially fold into a hairpin structure without large internal loops or bulges.

MiRNAs were found to play diversified roles from species to species [1,2]. In recent years, researches on the roles of miRNAs in cancers have been increasing tremendously, and miRNAs are suggested to have important therapeutic potential in human diseases. To date, there are 678, 472 and 287 miRNA entries for the human, mouse and rat genomes deposited in miRBase [3,4], the home of miRNA data on the web, in Release 11.0, respectively. Yet some studies suggested that the total number of miRNAs existing in a vertebrate genome can reach at least 800 [5,6], therefore continual efforts should be made on locating and verifying the unknown miRNAs. A number of computational prediction methods and software tools have been developed over the years for this purpose [7], however the datasets adopted by the various prediction tools are different and older methods are usually outweighed by the newly developed ones in terms of specificity and sensitivity.

In this paper, we first describe and validate the clustering phenomenon of miRNAs in the human, mouse and rat genomes by computational means. We then develop a clustering-based approach to a selected software tool, ProMirII-g [8,9], which was launched in 2006, aiming to filter their false positive miRNA predictions.

## Results and discussion

### MiRNA clustering

Many miRNAs are found to be arranged in clusters [10], meaning that they are in close proximity with their neighboring miRNAs. MiRNAs located in the same cluster are usually co-regulated and co-expressed [11,12], and recent studies suggest that miRNA clusters play important biological roles in specific tissues or genomes. Examples include cell proliferation in human lung cancer tissues [13], latent and lytic replication of Kaposi's sarcoma-associated herpesvirus [14], testis development and spermatogenesis in primates [15].

In view of this, we believe that miRNA clustering can be used to assist the prediction of novel miRNAs, and here we analyze how this idea can be applied computationally.

### Analysis of miRNA clustering in the human, mouse and rat genomes

**Figure 1:**
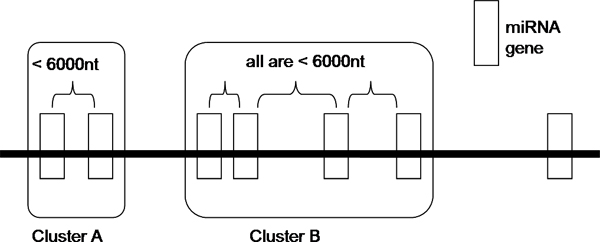
**Our definition of a cluster**. MiRNAs which are separated by a distance of less than 6000 nt are grouped as one cluster.

The definition of a miRNA cluster varies among researchers. Altuvia and colleagues defines a cluster in which there are two or more miRNA genes with pairwise chromosomal distances of at most 3000 nt [10]. Weber [16] suggested the following criteria of a cluster: same orientation and not separated by a transcription unit or a miRNA in the opposite orientation. A microarray study reveals that an abrupt transition in the correlation between pairs of expressed miRNAs occurs at a distance of 50 kb, implying that miRNAs separated by less than 50 kb typically derive from a common transcript [17]. In many other studies, the term 'cluster' is used without a proper and clear definition [18-20], and is also used to describe the phylogenetic relationships of miRNAs [11,21]. To assess the clustering property of miRNAs in the human, mouse and rat genomes, we want to have our own definition of a miRNA cluster. We define that two miRNAs belong to the same cluster if (1) they are located on the same strand of the same chromosome, i.e. same orientation; and (2) they are separated by a chromosomal distance of not more than 6000 nt. This distance of 6000 nt is not arbitrary. We first choose six different distances, which are 1500 nt, 3000 nt, 6000 nt, 10000 nt, 25000 nt and 50000 nt, and then we test the effect of the distances on the number of clusters and the number of clustered miRNAs formed. As shown in Table 1, there is an abrupt increase in the number of clustered miRNAs from the case of 3000 nt to the case of 6000 nt. There are little effects on the number of clustered miRNAs and the number of clusters defined when the separation is more than 10000 nt. To conclude, among the six distances that we have tested, 6000 nt is an optimal chromosomal distance bound within which two clustered miRNAs are separated. Figure 1 illustrates our definition of a miRNA cluster and Table 2 summarizes our results of the miRNA clustering analyses. The human and mouse datasets used in this paper were downloaded from Release 10.0 of miRBase and the rat datasets were from Release 10.1. There are 45.45%, 51.86% and 48.67% of the total miRNAs that are clustered in the human, mouse and rat genomes, respectively.

**Table 1:** Effects on the distance of chromosomal separation of clustered miRNAs on the number of clustered miRNAs found in the human, mouse and rat genomes.

**Genome**	**Effect**	***Chromosomal distance at which two clustered miRNAs are separated***					
		
		1500 nt	3000 nt	6000 nt	10000 nt	25000 nt	50000 nt
Human	# of clustered miRNAs	196	217	240	241	242	261
	
	# of clusters defined	71	68	60	60	60	65
	
	Average cluster size	2.76	3.19	4	4.02	4.03	4.02

Mouse	# of clustered miRNAs	204	215	237	243	253	260
	
	# of clusters defined	70	53	55	57	58	61
	
	Average cluster size	2.91	4.06	4.31	4.26	4.36	4.26

Rat	# of clustered miRNAs	119	129	145	154	154	160
	
	# of clusters defined	46	45	48	49	47	48
	
	Average cluster size	2.59	2.87	3.04	3.14	3.28	3.14

"Average cluster size" is equivalently to the average number of miRNAs found in a single cluster. It can be seen that there is an abrupt increase in the number of clustered miRNAs from the case of 3000 nt to the case of 6000 nt. There are little effects on the number of clustered miRNAs and the number of clusters defined when the separation is more than 10000 nt. To conclude, among the six distances that we have tested, 6000 nt is an optimal chromosomal distance bound within which two clustered miRNAs are separated.

**Table 2:** The number of clustered miRNAs and isolated miRNAs found in the human, mouse and rat genomes using our definition of miRNA cluster.

		**Human**	**Mouse**	**Rat**
Version of miRBase		10.0	10.0	10.1

Total # of clusters		60	55	48

Size of clusters	Min	2	2	2

	Mean	4	4.31	3.04

	Max	43	52	17

Isolated miRNAs	#	288	220	154

	%	54.55%	48.14%	51.33%

Clustered miRNAs	#	240	237	146

	%	45.45%	51.86%	48.67%

Total # of miRNAs		528	457	300

"Version of miRBase" denotes the update version of miRBase where the datasets are downloaded. "Size of clusters" is equivalently to the number of miRNAs found in a single cluster. There are 60, 55 and 47 clusters identified in the three genomes respectively, which are equivalent to 45.45%, 51.86% and 48.67% of the total human, mouse and rat miRNAs.

### Similarity analyses among clustered miRNAs, non-clustered miRNAs, neighboring sequences of clustered miRNAs and random sequences

As there are nearly half of the total miRNAs organized in clusters, we are interested in testing whether there are any relationships or similarities among them. We assess the sequence and secondary structure similarities among miRNAs in the same cluster by aligning the precursors of each clustered miRNA with the sequences from the following four categories in a pairwise manner:

(i) its fellow miRNAs found in the same cluster;

(ii) miRNAs located outside its cluster;

(iii) random sequences extracted from the genome; and

(iv) neighboring sequences extracted from its flanking 3000 nt regions.

**Figure 2:**
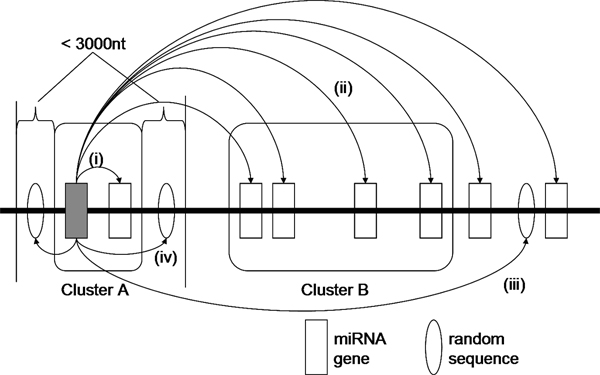
**Similarity analyses of a clustered miRNA with four groups of sequences**. A clustered miRNA is aligned with sequences from four categories: (i) miRNA(s) in the same cluster; (ii) miRNAs outside its cluster; (iii) random sequences extracted from the genome; and (iv) random sequences extracted from its flanking 3000 nt region.

The software *T-COFFEE *[22] (Version 5.05) is used for pairwise sequence alignment. The program *RNAdistance *of the Vienna RNA package [23] (Version 1.7.1) is used to compute the distance between two miRNA secondary structures, which are determined by *RNAfold*. Figure 2 shows how a clustered miRNA is aligned to sequences of the four categories.

As shown in Table 3, there is no statistically significant difference among the sequence alignment scores of the four categories (t-test, p-value < 0.05), suggesting that sequence similarity is unlikely to be useful for identifying clustered miRNAs. Interestingly, the distance between the secondary structures of miRNAs located in the same cluster is found to be much smaller than the distance obtained by comparing the structures of clustered miRNAs with the sequences from the other three categories (t-test, p-value < 0.0001). In other words, clustered miRNAs are structurally more similar to one another, and the *RNAdistance *score can be used to assess the structural similarity between two sequences. Based on this observation, we propose a clustering-based approach to improve the effectiveness of computational prediction of miRNAs.

**Table 3:** Results of the similarity analyses between clustered miRNAs and other sequences.

**Genome**		***T-COFFEE *sequence alignment score**				***RNAdistance *structure alignment score**			
Human	Category	Maximum	Average	Minimum	Std Dev	Maximum	Average	Minimum	Std Dev
	
	(i)	79	45.20	17	15.70	71	28.86	0	9.73
	
	(ii)	73	46.30	15	12.96	94	37.21	15	11.29
	
	(iii)	74	45.47	17	14.13	203	140.89	104	18.78
	
	(iv)	77	44.81	15	12.60	134	75.55	28	22.19

Mouse	(i)	79	43.63	0	14.40	72	30.41	0	9.33
	
	(ii)	78	45.28	16	13.03	69	35.19	10	9.67
	
	(iii)	75	43.72	16	14.40	239	139.93	103	17.31
	
	(iv)	72	45.37	15	13.33	128	69.55	26	19.31

Rat	(i)	75	44.28	17	12.83	113	31.41	11	12.60
	
	(ii)	72	45.18	16	13.12	116	35.68	13	11.73
	
	(iii)	69	45.03	16	12.48	198	143.12	110	18.43
	
	(iv)	73	47.90	15	13.26	114	74.69	32	18.74

Sequence and secondary structural alignments are performed for each clustered miRNA with sequences from the following categories: (i) clustered miRNAs, (ii) non-clustered miRNAs, (iii) random and (iv) neighboring sequences. A higher score implies a greater distance and hence a higher degree of dissimilarity. Std Dev, standard deviation.

### Performance analyses of ProMirII-g and miR-abela

We select two software tools to test our proposed clustering-based approach, namely ProMirII-g [8,9] and miR-abela [24]. In terms of positive predictive value (PPV) and sensitivity (SE), we first analyze the performances of these two prediction tools, and the results serve as a benchmark for comparison with our approach. Both ProMirII-g and miR-abela allow users to set a prediction threshold. Using a relaxed threshold, more true positives (TPs) and predictions will be obtained, yet at the same time a large number of false positives (FPs) will be included. In other words, a high SE and a low PPV are expected. Our approach aims at increasing the PPV by filtering as many as FPs as possible with the application of miRNA clustering.

Table 4 illustrates the results of the performance analyses. ProMirII-g works better than miR-abela on all the three genomes under our investigation, giving a SE ranging from 81.22% to 89.58%. miR-abela, on the contrary, does not show satisfactory performance on the prediction of human, mouse and rat miRNAs, with only around 60% of SE achieved. Both of them produce a large number of false FPs along with the TPs, and therefore low PPVs are resulted with a range from 13.31% to 31.16%. Clearly the prediction programs will be more useful and reliable if their FP rates can be reduced.

**Table 4:** Results of the performance analyses of ProMirII-g and miR-abela using human, mouse and rat genome data.

**Software**	**Statistics**	**Human**	**Mouse (All)**	**Mouse (Distinct)**	**Rat**
ProMirII-g	# of predictions	690	656	640	615
	
	# of TPs	215	199	183	127
	
	# of FPs	475	457	457	485
	
	# of real miRNAs missed	25	46	29	19
	
	SE	89.58%	81.22%	85.92%	86.99%
	
	PPV	31.16%	30.34%	28.59%	20.65%

miR-abela	# of predictions	1036	915	901	646
	
	# of TPs	149	140	126	86
	
	# of FPs	887	775	775	560
	
	# of real miRNAs missed	91	105	86	60
	
	SE	62.08%	57.14%	59.43%	58.90%
	
	PPV	14.38%	15.30%	13.98%	13.31%

It should be noted that in the mouse genome, 25 of the pre-miRNAs are duplicated. In other words, only 212 mouse pre-miRNAs are distinct in the genome. To avoid overestimation of the performance of the software, we identify the duplicated ones and conduct two measurements. #, number; FP, false positives; TP, true positives; SE, sensitivity; PPV, positive predictive value.

### Application of miRNA clustering: a clustering-based approach

**Figure 3:**
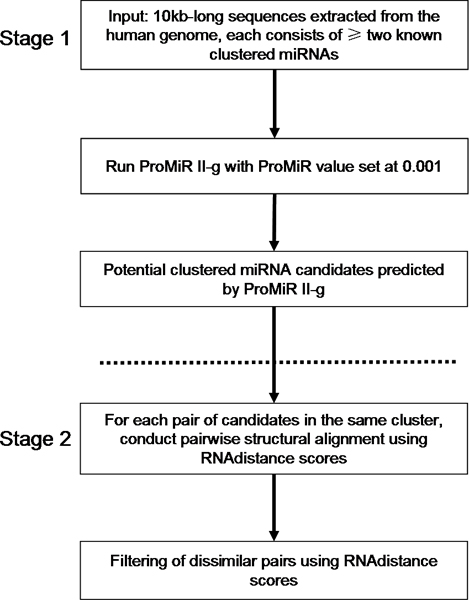
**Overview of the clustering-based approach**. In principle, our clustering-based approach consists of two stages. In stage 1, a currently available prediction program, for example ProMirII-g, is selected to produce a list of potential candidates. A loose threshold is used because we want to include as many TPs as possible to achieve a high SE. In stage 2, we aim at filtering the FPs from the list of candidates by picking out the dissimilar pairs as determined by the RNAdistance scores.

Recalling that clustered miRNAs are more structurally similar to one another as determined by the *RNAdistance *scores, we therefore design a clustering-based approach which utilizes this observation to filter false positives. The detailed steps of our approach are described in the section of Methods under the sub-title of "Our clustering-based approach" and Figure 3 provides the overview of the approach.

From Table 5, it is clear that our approach is able to increase the PPV to a large extent, from 17.37% to 21.25%. At the same time, it retains most TPs with just a slight drop of less than 10% in SE when it is applied to the human and the mouse genome. The approach appears to sacrifice the SE for the increase in PPV when it is tested on the rat genome. Since our filtering approach is based on miRNA clustering, it works best when the cluster has more than one TP. Table 6 shows a more suitable comparison when the clusters with less than two TPs are excluded from our test. The increase in PPV outweighs the drop in SE in all the three cases, suggesting that our approach is effective in filtering FPs without losing too many TPs.

**Table 5:** Results of our clustering-based approach on the human, mouse and rat genome data.

	**Human**	**Mouse (All)**	**Mouse (Distinct)**	**Rat**
# of predictions	374	381	359	276

# of TPs	196	172	165	101

# of real miRNAs	240	245	212	146

SE	81.67%	70.20%	77.83%	69.18%

Change in SE	-7.91%	-11.02%	-8.09%	-17.81%

PPV	52.41%	45.14%	45.96%	36.59%

Change in PPV	+21.25%	+14.81%	+17.37%	+15.94%

This table shows the SE and PPV obtained after our filtering approach is applied to the predicted candidates generated by ProMirII-g. The PPV is increased by more than 15% in all the three genomes. The SE is kept reasonably high as it is just slightly decreased by a percentage less than 10% in the human and mouse genomes.

**Table 6:** Results of our clustering-based approach when applied on clusters with more than one TP.

	**Human**		**Mouse (All)**		**Mouse (Distinct)**		**Rat**	
Before/After filtering	before	after	before	after	before	after	before	after

# of predictions	620	335	632	363	616	350	525	224

# of TPs	206	189	195	168	179	155	121	99

# of real miRNAs	220	220	234	234	202	202	132	132

SE	93.64%	85.91%	83.33%	71.79%	88.61%	76.73%	91.67%	75.00%

Change in SE	-	-7.73%	-	-11.54%	-	-11.88%	-	-16.67%

PPV	33.23%	56.42%	30.85%	46.28%	29.06%	44.29%	23.05%	44.20%

Change in PPV	-	+23.19%	-	+15.43%	-	+15.23%	-	+21.15%

If we exclude the clusters which bear no TPs or just one TP among the candidates predicted by ProMirII-g, we can see a great improvement in PPV without a significant effect on SE after our filtering approach is applied. The results agree with the principle of our approach, which is developed based on the phenomenon of miRNA clustering. In other words, if there are no clustered miRNAs in a sequence, our approach is not going to work properly. This table presents the results of a fairer comparison, suggesting that our approach is effective in filtering FPs.

## Conclusion

In this paper, we first validate the phenomenon of miRNA clustering in the human, mouse and rat genomes and confirm that there are more than 45% of the miRNAs in these genomes which can form clusters. We demonstrate that the secondary structure of a clustered pre-miRNA is more similar to its neighbouring pre-miRNAs located in the same cluster, when compared to the sequences outside clusters. Using this property, we design a clustering-based approach to filter the FPs resulting from a miRNA prediction software named ProMirII-g and successfully raise the PPV by a considerable amount ranging from 15.23% to 23.19% in the human, mouse and rat genomes. At the same time, the approach is able to retain a reasonably high SE. In view of this, we conclude that our approach is shown to be effective in raising the PPV of a software tool, particularly in the human genomes. We believe that it is worthwhile to carry out further experiments and tests with our approach using data from other genomes and other prediction software tools. Better results may be achieved with fine-tuning of parameters.

## Methods

### Performance analyses of ProMirII-g and miR-abela

The following steps are applied to data from the genomes of human, mouse and rat respectively.

Step 1: A group of 10000 nt-long sequences are extracted from the genome as the input sequences to the prediction software. Each of the long sequences consists of the clustered miRNAs identified in the genomes as mentioned in the section of "Analysis of miRNA clustering in the human, mouse and rat genomes". Large clusters which span over 10000 nt are split into smaller clusters.

Step 2: Each sequence is inputted to ProMirII-g and miR-abela. For ProMirII-g, 0.001 is selected as the prediction threshold (ProMiR value). For miR-abela, the prediction threshold is set at -10. Other parameters are set by default. A list of outputs, representing the potential miRNA candidates, is generated.

Step 3: The outputs are checked against the clustered miRNAs found in the genomes. The output candidates which match the clustered miRNAs are the true positives, and the rest of the predicted candidates are the false positives.

With the total number of predictions, the total number of clustered miRNAs in the genome, the number of TPs and the number of FPs, we evaluate the performance of the two miRNA prediction tools in terms of the SE and PPV. The formulas for calculating SE and PPV are as follows:

SE = TP/total number of clustered miRNAs in the genome

PPV = TP/total number of predictions, i.e. TP/(TP + FP)

Table 4 summarizes the results.

### Our clustering-based approach

#### Stage 1

A miRNA prediction program with prediction threshold set at a relaxed value is run with the same set of input sequences as described in the performance analyses of the prediction software. A list of candidates is produced, which are potential clustered miRNAs. Since the performance of miR-abela is not satisfactory, only ProMirII-g is used to test the effectiveness of our approach. 0.001 is chosen as the predictive value as it is the most relaxed threshold.

#### Stage 2

Pairwise structural alignment between each pair of candidates is conducted using *RNAdistance*. *RNAdistance *reads RNA secondary structures and calculates one or more measures for their dissimilarity, based on tree or string editing (alignment). Briefly, it first translates the RNA secondary structures, which is inputted by the user using the bracket format or coarse grained representations, into tree structures. The standard morphologic features like bulge, internal, multi-branch and hairpin loops are captured in the tree structures. It then aligns the trees using a multiple alignment program [25]. Since a higher *RNAdistance *score implies that the pair of candidate sequences have relatively different structures and vice versa, if a candidate has high pairwise *RNAdistance *scores with other candidates, it is likely to be a false positive and should be eliminated. The crucial step lies on how to determine candidate(s) with high scores and filtered them from the results. After several trials, we propose the following steps to do the filtering:

#### Step 1

Calculate the lower quartile (LQ) score of all the pairwise *RNAdistance *scores formed by the candidates as the threshold. If a cluster has less than four candidates, the average score will be taken as the threshold.

#### Step 2

Select the potential candidates. Potential candidates are candidates which can form a pairwise score less than the threshold with another candidate, and these two candidates are regarded as a linked pair. For example, given that c1 and c2 are two of the candidates and c_{1, 2} denotes the *RNAdistance *score when they are aligned to each other, if the threshold score is 40 and c_{1, 2} is 32, c1 and c2 are potential candidates and linked pair.

#### Step 3

Adopt a brute-force approach and enumerate all combinations formed by the potential candidates with replacement. Only the combinations that are formed by linked pairs are of our interest. For example, if there is a combination of c1, c2, c3, c4 and c5, but c1 and c5 does not form a linked pair (i.e. c_{1, 5} is less than the threshold), this combination will be discarded. Each combination is given a "R score" which is calculated as follows:

R_{1, 2,..., *k*-1, *k*} = (1/*n*)(sum(c_{1, 2}, c_{1, 3}, c_{1, 4}, c_{1, *k*}, c_{2, 3}, c_{2, 4}, c_{2, *k*},...., c_{*k*-1, *k*})), where *k *is the total number of candidates in the combination and *n *is the combinatorial *k*C2 = *k*(*k*-1)/2

In simple terms, R score is calculated as the average of all the pairwise *RNAdistance *scores formed by the candidate pairs in the combination.

#### Step 4

Another threshold has to be determined using the R scores in order to select the final candidates from the combinations formed in Step 3.

If there is only one combination formed, the candidates which form this combination are taken as the results.

The R scores will be sorted in ascending order, e.g. R1, R2, R3, R4,...., R*k*, where *k *denotes the total number of combinations formed in Step 3. If *k *is bigger than or equal to 3 and (R3 – R2) < (R2 – R1), the threshold is taken as R3. Otherwise the threshold is taken as R2. If the threshold is less than 30, the threshold is set at 30. Candidates which form a combination with a R score less than or equal to this threshold are the TPs and will be outputted as the answers.

## List of abbreviations used

FP(s): false positive(s); LQ: lower quartile; miRNA(s): microRNA(s); nt: nucleotides; PPV: positive predictive value; SE: sensitivity; TP(s): true positive(s).

## Competing interests

The authors declare that they have no competing interests.

## Authors' contributions

WSL carried out the above studies and analyses, participated in the design of the study and drafted the manuscript. MCML and DWC revised the manuscript for important intellectual content. SMY participated in the design of the study and revised the manuscript. All authors read and approved the final manuscript.
